# Nude Bodies in British Women’s Magazines at the Turn of the 1970s: Agency, Spectatorship, and the Sexual Revolution

**DOI:** 10.1093/shm/hkac032

**Published:** 2022-08-11

**Authors:** Daisy Payling, Tracey Loughran

**Affiliations:** Department of History, University of Essex, Colchester, CO4 3SQ, UK; Professor of Modern History, Department of History, University of Essex, Colchester, CO4 3SQ, UK

**Keywords:** sexual knowledge, sexual revolution, women’s liberation, nudity, advertising

## Abstract

Around the turn of the 1970s, women’s magazines began to feature naked female bodies in advertisements for health and beauty products. By the mid-1970s, this nudity had largely disappeared. This article examines the reasons for this spike in nude images, the types of nakedness depicted, and what this tells us about prevalent attitudes to femininity, sexuality and women’s ‘liberation’. Focusing on representations of naked female bodies allows us to explore definitions and operations of sexual ‘knowledge’, especially the role of mass media sources in influencing inchoate ideas about sex and sexuality. In this way, we consider the complex interaction between representation and experience in constructions of sexual knowledge, challenge theories placing women as passive objects of the male gaze and nuance notions of female agency in ‘sexual revolution’.

## Introduction

Towards the end of the 1960s, images of nude and semi-nude female bodies started to proliferate on the pages of conservative mass-market women’s weekly magazines, as well as those of their more daring monthly competitors. These images appeared in all types of content, but were most prominent in advertisements for diverse products, including underwear, tights, slimming aids, bust developers, deodorants, bath foams, shampoo, moisturisers and tanning oils. All conscripted naked female bodies to the consumerist cause, but framed these bodies differently: clean, sterile and hygienic; ‘natural’, liberated and free; dreamy, sensual and romantic; or glamorous, confident and hyper-sexualised. Some depicted women as active erotic agents, even directly co-opting feminist discourses of liberation, while others adopted a voyeuristic perspective, showing unclothed and apparently unaware women in private spaces. All, however, objectified the naked female body, and did so in a mass-market medium aimed at women. Such ads appeared in virtually every issue of these magazines until the early 1970s, when their frequency dropped off. For a short time, in a world where sex education was patchy and central heating was a luxury, women who might never deliberately look at their own nakedness could not pick up a magazine for recipe tips or knitting patterns without seeing nude female bodies.

This article considers the contents, contexts and implications of this explosion of nudity in mass-market women’s magazines between 1968 and 1972, when these images were most prominent. Our analysis centres on ads for vaginal deodorants, bath foams and oils and tanning products. These products straddle the line between health and beauty, and illustrate different themes found across the full spectrum of nude and semi-nude advertising, including attempts to stoke anxiety or stimulate longing, emphasis on care of the self and attention to effects on others. Our discussion highlights particular features of marketing for each product type, common features across advertising for different products, the underlying assumptions about femininity these reveal and the degree to which ads sexualised the nude body or deployed nakedness for other purposes. We draw on ads printed in the weekly magazines for housewives, *Woman* and *Woman’s Own*, and the monthly publications aimed at the ‘liberated’ woman, *Nova* and *She*. This approach allows us to identify the replication of specific images and reiteration of similar themes across differently-oriented magazines, and therefore to assess how nude images were deployed across the full sweep of mainstream women’s print culture.

This article has two related ambitions. First, we contribute to debates on the extent and consequences of the 1960s ‘sexual revolution’ by providing a woman-centred perspective on nudity and ‘permissiveness’ that focuses on mainstream culture. To date, the most influential scholarship on nudity in 1960s print culture is Marcus Collins’ exploration of the relationship between ‘permissive’ culture and the subcultural form of pornography.^1^ Collins argues that the late 1960s ‘pornography of permissiveness’ drew on tropes of liberation to portray women as actively desirous, but that male dominance and female submissiveness reappeared in the early 1970s. However, this rich analysis deals only with material produced by and for heterosexual men, within a format on the edges of mainstream culture. It does not aim to tell us about images produced for and consumed by heterosexual women in their everyday lives. By considering nudity in mass-market women’s magazines, and how women potentially and/or actually engaged with such images, we offer an alternative perspective on the interrelation of gender, sexualisation and ‘permissiveness’ in British culture at the turn of the 1970s. This examination helps us to understand the positioning of heterosexual women within the ‘sexual revolution’, especially at the intersections of gender, sexuality and whiteness. Although nude female bodies were deployed in different ways, with the aim of provoking different kinds of responses, without exception the bodies in our sample of ads were young, slim and white.^2^ Thinking about what is absent from these images, as well as what is present, opens up questions around power/lessness that require investigation beyond the parameters of this article.

Our choice of the descriptive terms ‘nude’ and ‘naked’ reflects this interest in probing the power/lessness divide. Within art history and criticism, there is a long-standing distinction between ‘nakedness’ and ‘nudity’. In his 1956 study of the nude, Kenneth Clark distinguished between nakedness as a state of powerlessness, vulnerability and embarrassment, and nudity as a ‘balanced, prosperous and confident’ aesthetic.^3^ John Berger adopted and modified this analysis, equating nakedness/authentic selfhood and nudity/display, while emphasising that both states represented lived sexuality.^4^ More recently, Philippa Levine has shown that in 19th-century images of British colonial subjects, ‘nakedness, as distinct from the nudity inherited from the classical tradition’ increasingly symbolised ‘colonial primitiveness, savagery and inferiority’. In contrast to fraught debates on the decency of displaying naked white human forms in high art, the circulation of images of colonial and non-white people caused no furore. Anthropologists, naturalists, administrators and metropolitan publics saw non-white nakedness as ‘natural’; the proliferation of such images denied colonial subjects the privacy perceived as a European right, and testifies to unequal racialized power relations.^5^ In this article, we use ‘nakedness’ and ‘nudity’ interchangeably, not to deny the political and aesthetic distinctions of such scholarship, but to indicate both the complex status of advertising as a lowbrow form that nevertheless drew on high art tropes, and the intersecting hierarchies of power revealed in the context of magazines, where white women’s nakedness demonstrated their lack of right to privacy, but non-white bodies were barely represented at all.^6^

Our second aim is to challenge prominent and still-influential theories that position women as passive spectators, and instead highlight their agentic capacities. The images women saw around them daily helped to form their sense of how they should act, what they could do and who they could be. Since the 1970s, feminist scholars have argued that mass-market women’s magazines played a crucial role in mediating messages about femininity and shaping female subjectivity. Through these magazines, women learnt about femininity and their place in the world. By exploring different ‘ways of seeing’ nudity, we consider how these images inflected women’s potential and/or actual bodily and sexual self-knowledge. In showing the multiple possibilities of spectatorship, we highlight women’s agentic capacities, in contrast to theories depicting them as powerless to negotiate patriarchially determined visual culture. This approach further generates the interrelated effects of questioning what constitutes sexual expertise and/or knowledge, emphasising the ambiguities of such knowledge for different groups (men/women, heterosexual women/queer women, white women/women of colour and so on), and opening out the possibilities of women’s manifold responses to nude imagery.

In line with the aims of this special issue, we consider mass-market magazines as sites of ‘sexpertise’ that contained multifaceted visual and textual representations of sexual subjects. The relation of these representations to sexual knowledge and expertise is not self-evident. Scholarship on ‘sexpertise’ has tended to focus on the production of sexual knowledge by largely elite experts, or the circulation of information about sex within and between different social groups. This special issue goes further by understanding sexpertise as comprising claims about sex that reject the notion of sexual behaviour as belonging to private, rarefied or confidential domains, and instead perceive it as a form of knowledge constituted within (and by) the public realm in mass societies. Certainly, mass-market magazines contributed to the formation of sexpertise in this sense. Our final section, which places the nude bodies of advertising against other content, indicates the multiple voices and formats discussing sex in mass-market women’s magazines. As they did so, magazines created both new forms of knowledge about sex, and new ‘experts’ deemed capable of speaking about sex—from the agony aunt, counselling those in need, to the reader herself, putting forward her own claims to knowledge on letters pages and in first-person features.

These voices are the backdrop rather than the focus of our exploration, however. Instead, we want to think through the power of images in mass-market magazines to shape women’s understanding of their own bodies, and their capacities for sexual choices and feelings, including different kinds of pleasures and shame. Historians still tend to think of ‘knowledge’ as bounded, formalised and easily locatable, and of ‘expertise’ as connoting full command of knowledge in this guise. But if we make publics and the public realm central to understandings of sexpertise, we must also create space for everyday knowledges created beyond text, often existing on the horizon of consciousness beyond full realisation or articulation, and for concepts of expertise that recognise the power of fluency in such all-pervasive, shifting and ill-defined everyday knowledges. This focus on non-textual forms of knowledge that permeate mass culture extends recent scholarship emphasising the rise of ‘ordinary’ expertise in the post-war period.^7^ It also, however, constitutes a distinctly feminised and queered perspective on sexpertise—one that rejects the implied primacy of rational, scientific and circumscribed forms of sexual knowledge and expertise, and instead embraces fluidity and ambiguity in ways of seeing, knowing and being. As such, it is an approach in keeping with our rejection of the ‘male gaze’ as a lens for viewing images of women’s nude bodies.

Our emphasis is therefore on the kinds of sexual ‘knowledge’ that circulated in semi-nude and nude images in advertising, and how readers may have reconstituted this sexual knowledge as it related to their embodied experience. This is no easy task, and it eludes certainties. It is notoriously difficult to trace the relationship between representation and experience, partly because the weight of surviving evidence tips so heavily towards static texts or images. Likewise, it is impossible to construct watertight arguments about what specific images ultimately ‘meant’ or how they shaped sexual subjectivities. Images are always open to multiple interpretations. Moreover, neither we nor our readers can help viewing the images of the kind discussed here through the prism of subsequent visual languages of sex—but, given the multiplicity of such languages, there is no guarantee that each viewer will see the same lights refracted. Indeed, where we see in some of these ads evidence of the slipperiness of the sensual and the sexual in female-centred imagery, our peer reviewers saw overt sexualisation.^8^ We wondered, with Catherine McCormack, whether their interpretation is because ‘images have taught us that female bodies experiencing pleasure must always—*can only—*be an erotic spectacle for a presumed gaze’.^9^ Consequently, we have let our original interpretations stand, but gesture towards these differences to reinforce a point of central importance: that we commit an injustice against our historical subjects when we insist ours is the only possible reading of their lives. Our conclusions, then, are necessarily speculative—a stance that reflects our respect for the lost and unrecorded world views of the women who read these magazines.

## Sexual ‘Revolution’, Sexual Pleasure and Bodily Self-Knowledge

The years from 1968 to 1972 saw visible tumult in gender and sexual relations. In 1968, the national press extensively covered the 50th anniversary of women’s partial admission to the franchise and the strike of female machinists at Ford’s Dagenham plant, alongside the rolling groundswell of activism that gathered into the Women’s Liberation Movement (WLM).^10^ Journalists variously lauded or lamented women’s increased socioeconomic power, new assertiveness, and sexual ‘liberation’, but rarely disputed that women had never had it so good. Often, these apparent gains were perceived as bound up with the onward march of ‘the permissive society’, as enshrined in liberalising legislation including the Sexual Offences Act (1967), the Abortion Act (1967) and the Divorce (Reform) Act (1969). Although the effects of ‘permissiveness’ were uneven, multidirectional and far from complete by the early 1970s, contemporaries certainly felt that they were living through a period of rapid social change, especially in regard to sexual mores.^11^ As the emphasis within mainstream culture moved towards sex as pleasurable rather than shameful, and as publishers and others pushed at the boundaries of the Obscene Publications Act (1959), what was permissible in the realm of cultural representation opened out.^12^ Hera Cook’s juxtaposition carries considerable force: ‘In 1960, pubic hair could not be legally shown in magazines. By 1970, naked actors were simulating sex on stage in *Oh! Calcutta!*’.^13^*Something* clearly shifted in gendered sexual attitudes and behaviour at this time, even if it is difficult to say exactly what.

The relevant question here is not whether there was a ‘sexual revolution’, but the extent to which heterosexual women shared in it, and in what capacity. As Ben Mechen reminds us, ‘liberalisation for some was not “liberation” for all’.^14^ It is easy to pit individual testimonies against each other: for every Angela Carter who believed that the sixties ‘changed, well, everything’ concerning sex for the better, we can find an Anna Coote and Beatrix Campbell to assert that the ‘permissive society’ ‘kidnapped [women] and carried them off as trophies, in the name of sexual freedom’.^15^ We can partially reconcile these opposing viewpoints by shifting focus from the totalising effects of sexual revolution to heterosexual women’s abilities to negotiate its perils and potential pleasures—abilities at least partly dependent on their sense of themselves, and their recognition by others, as autonomous beings with the same rights to and capacities for sexual enjoyment as heterosexual men.

The capacity for sexual self-actualisation is determined partly by individual experiences, and partly by socialisation. At the mid-century, girls and women were not socialised to understand much about their own bodies, or to actively pursue sexual pleasure. Opportunities to view other naked bodies were limited and site-specific, and many women felt shy about undressing even in front of their children.^16^ A ‘culture of sexual ignorance’, differentiated by gender and class, was pervasive in Britain until at least the 1950s.^17^ Men of all classes were expected to learn enough to be able ‘to guide their virginal brides through the wedding night’ without becoming ‘morally tainted’ themselves.^18^ Although middle-class and educated women tended to have more formal knowledge about sex than working-class women, women of all classes ‘consciously invest[ed] in the idea of female ignorance’ in order to maintain their innocence, respectability, and attractiveness.^19^ This culture of secrecy existed inside and outside the home. In the 1930s, only one-third of schools provided any sex education, and until the mid-1970s this often focused on the reproduction of animals. Women (not) educated in this tradition were ‘ignorant and ill-equipped’, found it difficult to discuss ‘taboo’ topics, and lacked the knowledge to better support their own daughters.^20^

The premium on female ignorance and passivity affected women’s sexual experiences throughout the 1960s and beyond. In her sex manual *Any Wife or Any Husband* (1951, reprinted 1962), Joan Malleson claimed that many women insisted on having sex in the dark, with husbands ‘never allowed to enjoy visual intimacy’.^21^ Two decades and one sexual revolution later, a culture of silence between heterosexual partners still persisted. In 1970, sociologist Ann Cartwright found that women preferred the pill to barrier methods of contraception because it allowed them to avoid touching their bodies, or discussing sex with their partners.^22^ As Chelsea Saxby’s research on 1970s self-help for cystitis has revealed, many women suffered for years because they had no language to discuss genitourinary pain, accepted pain as a ‘normal’ consequence of sex, or were not taken seriously by doctors.^23^ Writing in the 2000s, Jenny Diski argued that the sixties emphasis on casual sex as ‘part of the vital and present task of experiencing experience’ turned assent into a ‘contemporary version of good manners’, and that ‘not wanting’ sex was not seen as an adequate reason for women’s refusal.^24^

Following this statement, we might argue that women ‘brought up to accept, and prioritize, other people’s feelings, not to express their own feelings or desires’ did not understand sexual autonomy as their right; often, nor did the men with whom they had sex.^25^ Heterosexual women often perceived their own pleasure as secondary to their partner’s satisfaction, and this *de facto* relegation of their own sexual identities meant that certain kinds of questions around pleasure—indeed, certain kinds of conceptualisations of autonomous female sexuality—simply did not arise. The mid-century model of romantic love was a *companionate* sexualised ideal.^26^ Within it, male and female sexual pleasure were inextricably intertwined, but outside it, a model of independent male sexuality existed for which there was no female counterpart in Britain until the rise of *Cosmo* woman in the early 1970s.

This had ramifications for heterosexual relations. Women and men might perceive mutual sexual satisfaction as an important part of marriage and seek help if they could not achieve it. However, as Caroline Rusterholz has shown, many women also blamed themselves for their lack of sexual satisfaction and did not want to tell their husbands about it, because they valued his feelings over their own gratification. Likewise, male partners might believe female pleasure to be important, and earnestly seek to help their wives achieve it, but still believe that absence of orgasm indicated a fundamental flaw in the female make-up rather than a failure of their own technique.^27^ Along very different lines, Hannah Charnock has shown that during the era of ‘sexual revolution’ adolescent girls talked about sex and engaged in heterosexual activity for many reasons, including the desire for status within their friendship groups.^28^ Her discussion offers little indication that teenage girls’ *primary* reason for sexual activity, or their dominant framework for understanding why other girls had sex, related to the autonomous pursuit of pleasure. This further suggests that girls and women often lacked any sense of independent (hetero)sexual identity.

This gendered socialisation into bodily and sexual knowledge shaped women’s responses to nude imagery. By the end of 1970, when the *Sun* newspaper inaugurated its Page 3 tradition with a photograph of model Stephanie Rahn topless, female nudity was irrefutably acceptable in mainstream culture. The institution of Page 3 was the logical culmination of trends in visual culture over the previous two decades. Throughout the 1950s and 1960s, pin-ups had spread through the popular dailies and Sunday papers, while advertising more frequently pictured models in pin-up poses (no exposed nipples or bottoms).^29^ Nude and semi-nude female bodies were ‘increasingly integrated into a wider range of artistic and cultural genres’, especially cinema, ‘rather than being separated out as a specialized erotic or aesthetic category’.^30^ The expansion of British naturist periodicals in the same period is another aspect of this ‘continuum of models, photographers, and advertisers who traded in naked bodies as part of a rapid boom in commercial print culture and shifting patterns of taste’.^31^ In the 1960s, reportage on contemporary ‘permissiveness’ and the counterculture increasingly incorporated depictions of nudity, further normalising this imagery in mainstream contexts.^32^

As Marcus Collins has argued, pornography and ‘permissive’ culture also influenced each other. Against the madonna/whore dichotomy that implicitly structured earlier pornography, the ‘pornography of permissiveness’ personalised nude models and portrayed women as actively sexual. However, in the early 1970s more voyeuristic and depersonalised forms of pornography, emphasising male power and female passivity, or even glorifying violence, returned. This shift, which Collins views as part of a misogynistic backlash against the WLM, also coincided with the pornographication of the tabloid press.^33^ Yet even if sixties porn did depict more emancipated and desirous women, ultimately this material was produced by heterosexual men, for heterosexual men: it demonstrates a temporary and limited shift in marketable male fantasies, not in women’s expectations or behaviour, the representations targeted at female consumers, or even what heterosexual men necessarily demanded from girlfriends and wives. Nor did it really portray an autonomous female sexuality; the guiding principle of such pornographic literature was that ‘women nursed the same desires as men’ and that ‘women’s sexuality was now similar to men’s’.^34^ The assumptions of the ‘pornography of permissiveness’ might have spilled into the mainstream, especially in nudging the boundaries of acceptability further onwards, but there is little evidence that it altered how women were expected to act in their (unequal) negotiations with men.

In this charged context, the *Sun* institution of Page 3 further *‘*normalised female toplessness as mainstream popular culture’s primary symbol of sexual pleasure, and powerfully reinforced the idea that women’s bodies should be available for public scrutiny and consumption’.^35^ This opening up of women’s bodies to the (implicitly masculine) public gaze proceeded without commensurate increases in women’s bodily self-knowledge or autonomy. How, then, did girls and women raised in a culture of sexual silence, perhaps ill at ease with their own bodies and often unable to articulate their own needs, respond to the spike of nude and sexualised images in women’s magazines—in that small slice of mass culture designated the woman’s world?

## Ways of Seeing, Ways of Looking, Ways of Being

The question of how people internalise gendered representations is not new. In the 1970s, a body of scholarship emerged that still shapes discussions of women’s interactions with visual culture. These studies employed an active-male/passive-female binary, placed women as objects of the gaze (denying the possibility of man as spectacle), and assumed heterosexual spectatorship. John Berger’s *Ways of Seeing* (1972) argued that ‘*men act* and *women appear*. Men look at women. Women watch themselves being looked at’. Women were taught from earliest childhood to survey themselves constantly, with the act of self-objectification carried out by an internalised masculine consciousness.^36^ Berger’s view therefore saw women as incapable of any action outside of male spectatorship. Laura Mulvey’s 1975 article ‘Visual Pleasure and Narrative Cinema’ similarly depicted ‘Woman as Image, Man as Bearer of the Look.’ Women in films, ‘displayed for the gaze and enjoyment of men, the active controllers of the look’, served as ‘erotic objects’ for (male) characters within the screen story and (male) spectators within the auditorium alike.^37^ Both works influenced WLM analyses of how representations of women in mass culture contributed to women’s subordination. While the WLM emphasised women’s ultimate capacity to challenge masculinist culture, feminist writing in this tradition also often reinscribed the *current* status of women as ‘objects of the male gaze’ oppressed by male-controlled visual media.^38^

This scholarship recognised the always-gendered status of representation and spectatorship, the need for critical attention to mass cultural forms, and the influence of ‘everyday’ visual culture on subjectivity. However, it allowed little space for women to engage with existing visual culture as agents rather than passive victims, or for the possibility of multiple, contradictory and complexly gendered responses to images. In the 1980s and beyond, feminist critics explored alternative female responses to images, including identification and desire, and emphasised possible ways of viewing that resisted or subverted the intentions of producers.^39^ In 1989, Edward Snow argued that male gaze theory often became ‘an unwitting agent of the very forces of surveillance it wishes to oppose’, simultaneously reducing masculine vision ‘to the terms of power, violence, and control’, and depriving images of women of ‘their subjective or undecidable aspects—to say nothing of their power’. Snow proposed ‘resisting ideological (fore)closure’ and treating the very terms ‘male’ and ‘female’ as ‘subject to revision’.^40^ Queer theorists took up this invitation, conceptualising queer relations of looking, exploring contexts where patriarchal discourse did not completely determine women’s experience, and examining systems of fashion and advertising that encouraged pleasure in looking at eroticised images of other women/men.^41^

Here, we follow queer theory’s insistence on the multiplicity and indeterminacy of visual culture, gesturing towards the possibilities of these insights for understanding mass-market women’s media. This seems to us a more properly historicised approach to visual culture than much work on the male gaze. Crucially, Berger, Mulvey and WLM commentators did not simply analyse a visual culture; their critiques *emerged out of it*. This body of scholarship was generated at a specific historical moment when hyper-(hetero)sexualised images infiltrated television, newspapers and magazines. Assertions presented as timeless truths about visual culture, male–female relations and the gendered psyche were instead ‘time bound’; reflecting the profuse depictions of female bodies in mass culture at a point of shifting sexual relations—and replicating that culture’s blind spots around ‘race’ and queer sexualities.^42^ In reinstating the instability and final unknowability of women’s engagements with these images, we also insist on a fully historicised account of women’s agentic responses to this newly sexualised imagery; an account that acknowledges dramatic asymmetries of power and the difficulties in capturing the effects of such images on female subjectivity, but also avoids ahistorical statements about feminine consciousness or depicting women as inevitable victims of oppressive visual culture.

## Magazine Markets, ‘Permissiveness’ and Nudity in Advertising

The 1970s feminist scholarship on visual culture undoubtedly got one thing right: women’s magazines *were* an influential source of depictions of femininity. In 1964, over 50 million women read women’s weekly magazines, and around 34 million their monthly equivalents.^43^ These figures actually represented a decline from the late 1950s high point of sales. This troubled publishers, not least because it threatened relations with advertisers.^44^ Market research suggested that weeklies had failed to move with the times and were struggling to get to grips with ‘permissiveness’. As publishers experimented with wooing new readers, the editors of established weekly magazines aimed at housewives also tried to avoid alienating old ones. Their solution was to maintain much of the same content and visual style, while employing formats that emphasised direct communication and therefore implied a less hierarchical relationship with the reader.^45^ Increasingly, however, they competed for readers with newer, more self-consciously ‘liberated’ monthly titles. Publishers sought to boost sales by revamping old titles and creating new ones, including magazines aimed at the ‘new woman’—younger, educated women who had experienced the world of work. ^46^ Both types of magazine reflected and constructed women’s new social realities.

*Woman* and *Woman’s Own*, established in the 1930s, were ‘trade paper[s] for women at home’ offering advice on domestic matters such as choosing ‘new curtains for the living-room, a husband or a frock’, alongside a smattering of light entertainment.^47^ At the end of the 1960s, they were the top-selling women’s weeklies, with combined sales of nearly 5 million per issue, and circulation far beyond those who actually bought either publication.^48^ They were read by women across all age groups, social classes (with the highest percentage of readers among the wives of ‘skilled workers’), and areas of England, Wales and Scotland.^49^ They were read by men as well as women, but aimed squarely at a female readership.^50^

*She* and *Nova* were the most prominent mass-market women’s monthly magazines. *She*, launched in 1955 for women who were ‘funny, vulgar, and tough’, gained a reputation for outspokenness through novelties such as introducing British women to the concept of bidets.^51^ It tackled social and political issues, showcased adventurous pursuits like sailing alongside knitting patterns, and made space for ‘highly individualistic’ voices, whether sincere or irreverent.^52^ In 1965, it had a regular circulation of around 300,000.^53^ Although *She* spoke to women with interests beyond housewifery, the women who read it most likely were or expected to become housewives.^54^*Nova*, launched in 1965, described itself as a ‘new kind of magazine for a new kind of woman’—‘women who make up their own minds’, and who had ‘more to think about than what to do about dinner’.^55^ It was self-consciously aspirational, as reflected in its highly stylised aesthetic and dense think-pieces. In the late 1960s it reached a ‘highly educated or upper-middle class’ readership of c.160,000.^56^ Although their main target demographic was women, unlike the weeklies *She* and *Nova* both also sought to appeal to men.^57^

Until the late 1960s, none of these magazines regularly featured nudity. When nude and semi-nude images of women did begin to proliferate, it was not purely to titillate. Even *Nova*, the most highly sexualised magazine, presented such images as part of its unusual and challenging aesthetic. Nude and semi-nude images appeared much more often in advertisements than editorial content.^58^ Ads for a vast array of products featured nude and semi-nude bodies. In our sample of ads, these bodies were always young, slim and white. In the analysis that follows, we consider the (apparent) intention and audience of the ads versus the (potential) responses of actual readers. We show what kinds of images of female nudity women were exposed to in everyday life, situate these images within wider discourses around sexuality and femininity in women’s magazines, and explore women’s agentic capacities in interacting with these images. Against theories of the ‘gaze’ and spectatorship that present female responses to this imagery as uniform, and women as essentially passive in the face of misogynistic imagery, we emphasise the ambiguities within many images, and the multiplicity of possible responses to them.

Of course, the ultimate aim of ads was to sell the product. To this end, advertisers alternately played on women’s fears or incited their desires. A common technique was to portray uncontrolled and excessive ‘natural’ womanhood as potentially obnoxious and requiring continuous self-management (and therefore unending consumerism) in order to achieve an *appearance* of naturally desirable femininity. Alongside this contradictory construction of ‘naturalness’, the ads concurrently invoked and undermined the viewer’s own status as a ‘normal woman’. On the one hand, womanhood necessarily involved repellent excrescences of sweat, secretions and blood; on the other hand, women who did not continually strive to contain, mask and hide their leakiness would be marked out as abnormal and disgusting. Ads therefore simultaneously constructed womanhood as an inherently problematic state-of-being and individualised this problem. To entice women to resolve the ‘problem’ using their products, they depicted an alternative femininity, alluring because of its apparent ‘naturalness’ and ever-so-slightly glamorised ‘normality’. While these strategies undoubtedly pathologised elements of female experience, perversely, they also acknowledged aspects of embodiment that remained culturally taboo outside conversations between women; by representing shared bodily experience within the semi-private world of the women’s magazine, these ads also de-individualised that experience. In the context of wider cultural silence, these ads contributed to widening women’s bodily and sexual knowledge, even if the content of that knowledge was ambivalent and often highly problematic.

### Vaginal Deodorants

Ads for vaginal deodorants began to appear in women’s magazines in the late 1960s.^59^ As with ads for underarm deodorants, they appeared more frequently in the summer months. The volume of ads for vaginal deodorants peaked between 1968 and 1970, and fell off thereafter; likewise, nude models featured prominently over these 2 years, and after this point ads tended to show either clothed women or packaging. Across the entire period, ads for both underarm and vaginal deodorants stressed freshness, coolness, confidence and all-day protection. However, vaginal deodorant ads also highlighted women’s liability to unpleasant odours, the necessity of the product for sex and intimacy and sometimes notions of liberation. In their emphasis on shame, they moved away from notions of self-care and towards self-management for the sake of others, and especially lovers: as Sara Ahmed notes, one can only be shamed by someone ‘whose view “matters”’.^60^ In these ads, nudity hints at the complex and intertwined relationship of sexual desire and sexual shame, especially for women with little bodily or sexual knowledge who were expected to perform sexual liberation but were ill-equipped to do so.

Perhaps for this reason, to a far greater extent than ads for other products, those for vaginal deodorants consistently and explicitly posited femininity as both problem and solution. If ‘the most feminine part’ of a woman generated ‘odour and discomfort’, then only ‘the most feminine deodorant’ could solve the issue.^61^ The intertwining of notions of femininity, sexuality and shame is especially evident in ads that ran between 1968 and 1970. A 1968 ad for Elle vaginal deodorant (pictured below), a brand marketed at teenagers, drew on prominent tropes of Continental sexual freedom, and promised to tell ‘The naked truth about French girls’.^62^ The aerial photograph shows a young white woman, potentially a teenage ‘girl’, lying naked on a bed of petals. She is curled into a ball on her side with her hair hiding her face, arms hiding her chest and outer thighs hiding her genitals. The image shows a vulnerable young woman, while the textual repetition of ‘feminine’ underlines femininity itself as an at-risk state: ‘Elle is […] the most feminine word in the French language’, while the deodorant is ‘a feminine essential in any language’, and the ‘price of complete feminine protection’. Subsequent (non-nude) Elle ads heightened this emphasis, asserting that the ‘basic feminine deodorant problem begins where you are most basically feminine’, and that the reader would ‘hate [her]self’ if she did not use the product.^63^

**Figure:**
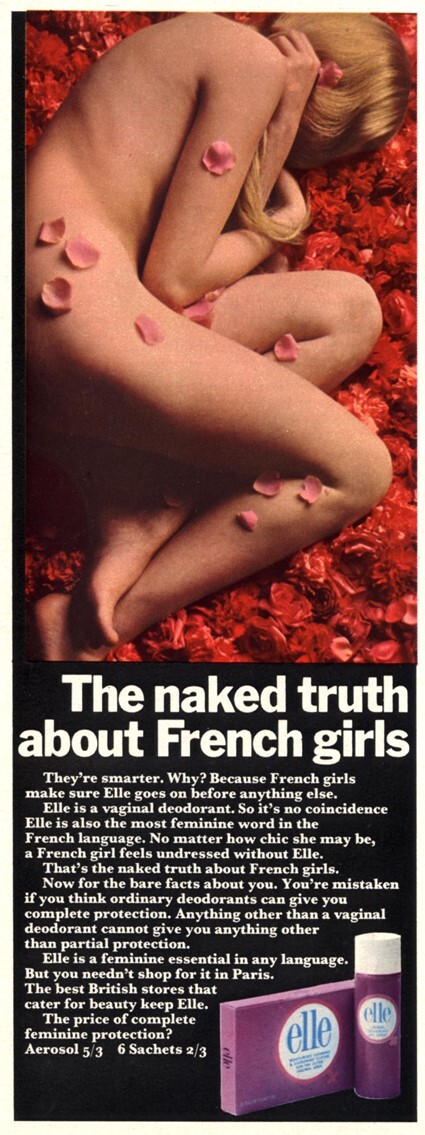
Image courtesy of The Advertising Archives

Ads for Bidex also used nudity to incite shame. A 1968 ad showed, from behind, a naked body from shoulder to knee, with the strapline across the buttocks reading ‘The deodorant nobody thinks they need’. Smaller text assured readers that the product would keep them ‘absolutely safe from embarrassing vaginal odour’.^64^ In another 1970 Bidex ad, the visual imagery carried the burden of demonstrating discomfort and humiliation. Some variants of this ad stated that ‘only Bidex has the patent on all-day comfort and confidence’, while others reassured that Bidex ‘doesn’t just cover up odour. It prevents it’. The accompanying image showed a naked young white woman perched on the edge of a seat hidden by flowers, representing the floral scent of the deodorant.^65^ The model turns her body away from the camera, but her breast and nipple are clearly visible in profile. A mirror shows the back of her head as she turns away from her own reflected body, her downcast face refusing to meet the camera’s gaze. The model’s nudity reflects the intimacy of the product, while her lowered head and eyes signify shame and alienation from her own body.^66^ The ad claimed that Bidex would ‘see you confidently through the day’, but the model’s body language showed uncertainty and discomfort.

From 1970, ads emphasised nudity and shame in increasingly (hetero)sexualised ways. An FDS ad (reproduced below) showed a naked white heterosexual couple from the waist up. The woman leans her head on the man’s chest and looks away from the camera. Her unsmiling mouth hints at concerns about ‘What a girl’s best boyfriend won’t tell her’.^67^ Throughout the twentieth century, ads for personal hygiene products often played on the notion that individuals were unaware of their own smell, but ‘politeness’ prevented other people from telling them.^68^ FDS went further by explicitly provoking anxieties about sex, intimacy and smell. The text spoke directly to the reader: ‘maybe you think you haven’t the problem. Perhaps you don’t. But it’s surprising how many girls do’. Of course, ‘we only mention it to you, because we wouldn’t want anyone else to’. Different versions of the ad followed this claim with ‘Especially your husband’ or ‘Especially someone you love’.^69^ Allusions to boyfriends, husbands and loved ones played on women’s fears that without vaginal deodorant they would be sexually undesirable. A 1970 Elle ad, also showing a naked white heterosexual couple embracing, explained that ‘excitement, anxiety, or your natural bodily functions can all cause odour’, in this way sexualising the product. Elle promised a ‘nicer way to be sure of yourself’ in implied sexual activity.^70^ Other non-nude ads warned that ‘if you want him to go on thinking about you in the nicest possible way you’d better go on using Elle’, or associated the ‘really feminine’ user of vaginal deodorants with ‘success’ in the marriage market.^71^

**Figure:**
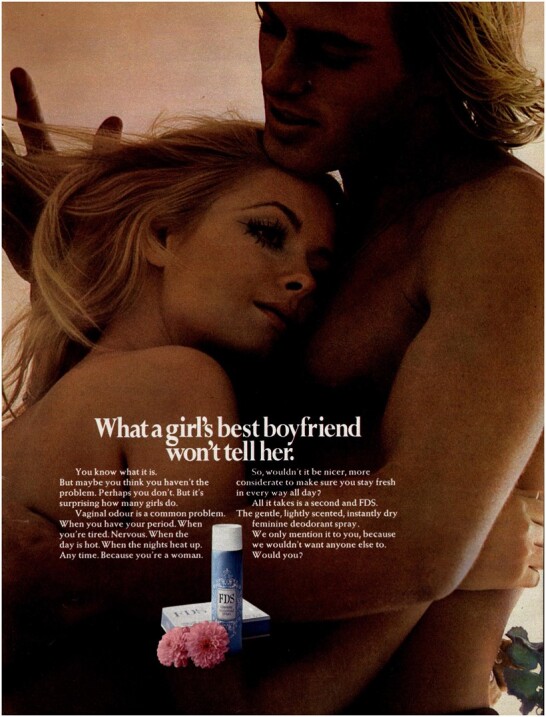
Image courtesy of The Advertising Archives

Although they ultimately preyed on the same fears about bodily odour, other ads instead celebrated femininity. A 1971 Femfresh ad showed a young white woman naked in the forest, foliage creeping up to hip height, running her hands through her hair.^72^ Head tilted up and away from the camera, she bares all. The accompanying text evoked modern working womanhood, a theme pursued in subsequent Femfresh ads, stating that ‘today’s girl knows that with her body working for her every moment, there will naturally be times when this could lead to odour and discomfort’.^73^ The text and image created an exhilarating picture of ‘feeling glad to be alive, glad to be a woman’, and Femfresh itself as ‘what feeling feminine is all about’.^74^ A later Femfresh ad, showing a very young white woman sitting with legs apart, staring straight at the camera and smiling, used rhetoric around adulthood to encourage teenage girls to use the product. Split down the centre from head to toe, the model is half-dressed in school uniform and half-naked. One breast and hip are fully displayed, but while the camera looks up her skirt, shadows hide her genitals.^75^ The text explained that ‘the more woman you are, the more you need Femfresh’, explicitly linking the deodorant with womanhood.^76^

Ads presenting vaginal odour as a ‘normal’ and easily resolvable problem in some ways represented an advance on earlier ads that deliberately incited shame. However, they also demonstrate the new assumption that women should integrate vaginal deodorants into their everyday hygiene regimes, as simply another ‘part of your make-up’.^77^ Until 1970, vaginal deodorant ads suggested that use was only necessary in specific circumstances, for example ‘after strenuous physical exercise, at times of nervous stress, in warm weather, when travelling, during menstruation, or at any time when you feel that bathing is not enough’.^78^ Some brands echoed the language of ‘problem days’ or feeling confident ‘every day of the month’ familiar from menstrual advertising, perhaps even showing menstrual products to underline the link.^79^ From 1970, however, most brands started to insist that vaginal deodorants should be used every day ‘as part of your daily hygiene’ to provide ‘everyday confidence’.^80^ This shift coincided with a relative decline in the frequency of nudity in ads, epitomised by the ‘Who uses Bidex? Busy mums like us’ campaign, which showed busy women of different ages going about their days working, raising children and seeing friends (and it is perhaps noteworthy that as women in such ads get older, their clothes go back on).^81^ Such ads portrayed vaginal odour as ‘a normal bodily function, but nevertheless a source of embarrassment’.^82^ The move from nude to clothed images, and from explicit shaming towards the ‘normalisation’ of vaginal odour, was not a straightforward transition towards a heathier and less objectifying approach towards women’s bodies. Instead, it showed how consumer brands could increase profit by convincing women to use products daily rather than for one week of the month.

### Bathing

In contrast to those for vaginal deodorants, ads for both bathing and tanning products that featured nudity placed much more emphasis on sensuality and pleasure. Ads for both types of products often invoked notions of distant climes, whether warm Mediterranean shores or bracing Nordic spas, at a time when international travel was becoming more common.^83^ In the 1960s, holidays abroad were ‘still something extraordinary’ for the majority of British people, but the end of the decade saw the ‘transformative’ effect of the package holiday industry which brought the glamour and luxury of foreign travel to many more people.^84^ By 1972, package holidays to Western European destinations had developed a reputation for offering ‘sun, sea, sand and sex’ to the masses.^85^ These ads therefore set up pleasurable associations between nudity, ease and leisure, while also referencing close-to-home or ambiguously Europeanised exoticism. However, appeals to continental pleasures were more prevalent in ads for tanning products, not only because of the clear link between suntans and holidays, but because in ads for bath oils and foams, an additional context led to emphasis on the interior world of the home: the late 1960s bathroom revolution.

At the start of the 1960s, 15 million Britons lived in homes without baths; many people still washed at sinks, filling a tub with heated water for baths once or twice a week; and in some areas up to one-fifth of the population had no hot water tap at all. By 1971, however, only 12.5 per cent of households lacked either an inside toilet, kitchen sink, hand wash basin or bath or shower with hot and cold water supply.^86^ Invocations of luxury and relaxation in ads for bathing products tapped into a newfound freedom to bathe in the nude in private and at leisure. Nude female models, gloriously alone while bathing (still the fantasy of many mothers of young children), displayed the luxury status of these items for many readers. The accompanying text framed this nudity to appeal to wider notions of sexual freedom and romance. The nudity in these ads was obviously context-specific—people used such products when naked—but the evocation of sensuality and sexuality speaks to a specific historical moment. The emphasis in such ads on sensuality also simultaneously warns us against viewing adult nudity as always and inevitably sexual, and encourages an understanding of sexual*ised* pleasure that neither relegates voluptuous delight in the self nor equates it with the mechanics of auto-eroticism.

Ads for bath foams and oils featuring nudity usually appealed to either the product’s refreshing and invigorating qualities, or its promise of lazy sensuality. In the former camp, a 1970 ad for Norsebad bath oil showed a young white blonde woman emerging naked from a bathtub apparently located in the middle of a forest of conifers.^87^ Arms raised, utterly unbothered by her nakedness, soap running down her torso, she beams at the camera. The model’s exuberance is further underlined by the strapline’s shout, ‘Hello Norsebad!’, and the text’s sign-off, ‘Goodbye, dull times! Hello, Norsebad!’. The copy encouraged the reader to ‘abandon’ herself in the ‘clear, fjord-green water’, and to ‘[s]et yourself free … and really come alive’. This ‘luxury herbal bath created in Scandinavia’ took every indulgence to the next level: not just luxury, but ‘scandalous luxury’, not just perfumed, but with a ‘seductively tangy fragrance’. It promised to cleanse, awaken and tone, rather than to relax the user. Norsebad used nudity to express the invigorating liberation of being at one with nature and naked in the wild and tapped into notions of health-giving northern European spa culture and sexual freedoms.^88^

Other ads represented bathing as a private moment of self-indulgent relaxation. They showed models submerged in tantalisingly blue pools, often with arms wrapped around their chests, a classic nude pose to hide breasts, but here suggesting the woman caressing herself. A 1969 ad for Revlon’s Moon Drops bath oils showed a young white woman relaxing in a pool, called on readers to ‘take the naked plunge’ in water emulating a ‘natural hot spring spa’, and emphasised the appeal of the ‘private, naked life’.^89^ Similarly, a 1968 Fenjal ad (reproduced below) showed a naked white woman half-submerged, her lower half blurred but clearly naked beneath the water, and the arrangement of her limbs perhaps obliquely referencing the classic pose of Venus in western art.^90^ The jewelled alice band on her long hair accentuated her bodily nakedness while adding a hint of glamour. The ad, headed ‘The Fenjal Touch’ drew the reader into the experience of closing the bathroom door, leaving the noisy world outside, and ‘[s]wirling lazily into dreams’ with ‘[s]ilky-blue Mediterranean Fenjal’. Other late 1960s Fenjal ads developed these themes. One appealed to ‘the girl who’s lazy about moisturising her skin’, with copy repeatedly invoking the pleasures of languorous relaxation.^91^ Another further emphasised the almost orgasmic pleasures of ‘the unique Fenjal touch’, the only brand that ‘can cream your body to lasting softness’.^92^ A 1970 Escapade ad added ‘rapture and romance’ to this heady mix.^93^ It showed a young white woman standing naked in an intensely vivid blue pool. She embraces herself, hiding her naked chest as her gaze tilts away from the camera. The accompanying text introduced the idea of an evening bath and cast itself as the woman’s lover: ‘Surround yourself with Escapade tonight and surrender to its charms […] Ecstasy begins with Escapade’.

**Figure:**
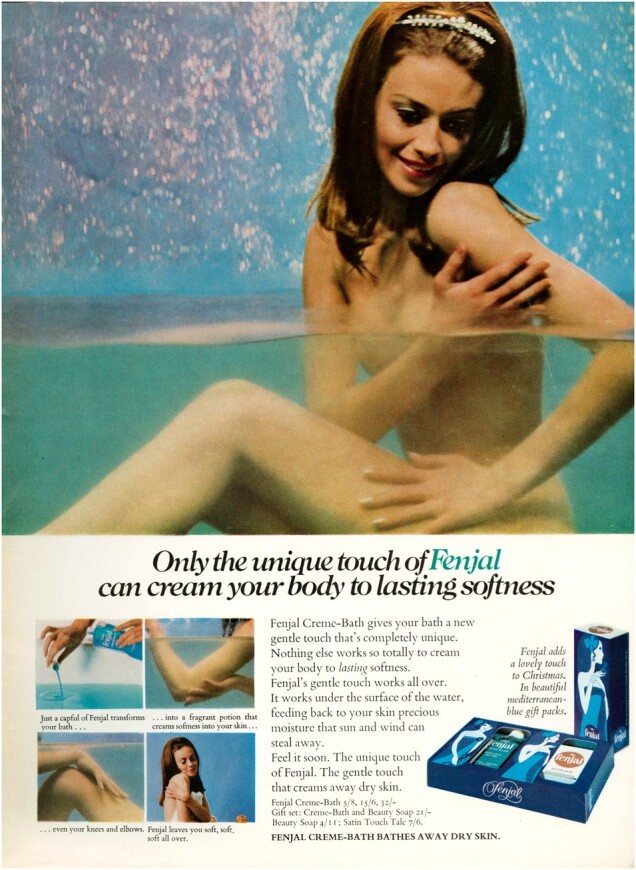
Image courtesy of The Advertising Archives

Ads using the language of romance and showing women alone, caressing their skin, hinted at voyeurism. This hint deepened in a Mornay Bath Collection ad with a slightly blurry image, as if seen behind a wet shower screen, of a naked white model with arms crossed over her chest, touching her neck.^94^ Although the woman is standing, the text described baths as ‘a big, warm, scented heaven of luxuriant luxury’, and promised to ‘turn your bathroom into a most ungrimm modern fairytale’ called ‘BEAUTY & THE BATH’. The bath stands in for the beast, Belle’s eventual lover, while the framing of the image as a fairy-tale turns the model into an actor in a story. Such ads sat alongside the romantic fiction that formed a staple of mass-market women’s magazines, itself drawing on the popularity of romance novels with women readers of different ages and socioeconomic backgrounds.^95^

A series of ads for Badedas bath oils that ran between 1970 and 1973 expanded these themes, moving fully into the sphere of sex and romance.^96^ The strapline ‘Things happen after a Badedas bath’ implied that the bath oil created the mood for sex. The ads (an illustrative example is reproduced below) showed a white woman holding a towel to her chest, gazing at an approaching man. Shot from behind, we see her bare back. The towel swoops low exposing the top of her bottom and water droplets glisten on her shoulders. In most ads, she stares through a window at an approaching man; in one exception, she stands in the corridor of a stately home, with a man in Hussar’s uniform standing at a distance in front of her, drawing on the tropes of popular Regency romances.^97^ In other ads, the approaching man vaults a wall into a garden, rows a boat across a lake framed by mountains and a castle, stares intently in a sharp suit or stands by a sleek and expensive-looking car. The ads invite women to imagine ‘being cleansed more kindly, more gently that you’d believe possible’, but conclude that ‘it’s what happens afterwards that matters’. These ads complicate the simplistic notions of active male and passive female underlying theories of the male gaze. The product made women sexually desirable and drew men to them, but the adverts also encouraged women to fantasise, and bring a broad cast of players to bath-time. The narratives of these ads invited readers to complete them using their own imaginations, and demonstrate advertisers’ awareness of consumers as active participants in creating stories of romance, pleasure and glamour—their awareness, we might say, of a female gaze looking back at the page.

**Figure:**
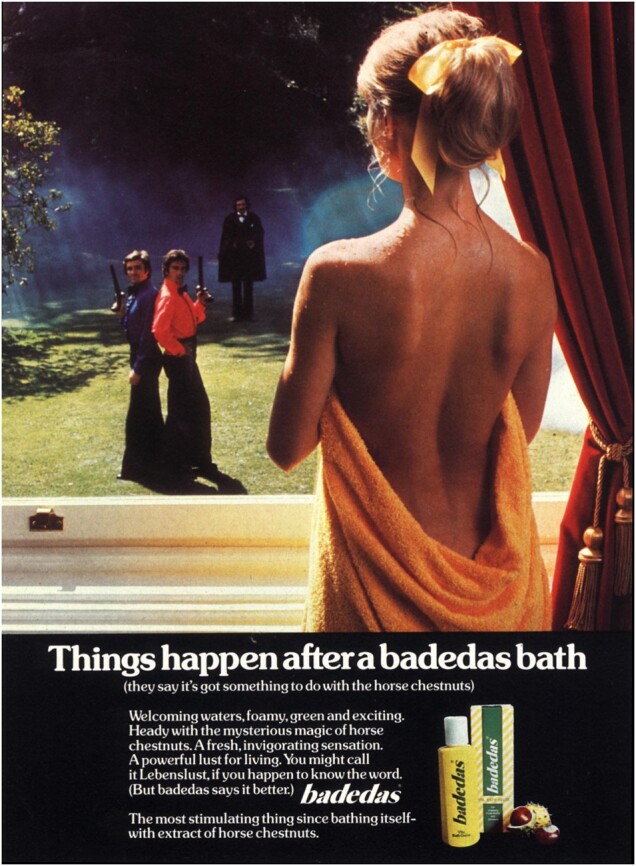
Image courtesy of The Advertising Archives

### Tanning

In their invocation of sensual pleasure, ads for tanning products had much in common with those for bath foams and oils. Ads for tan-boosting make-up, fake tan, tanning oil and moisturising after-sun creams did not change very much over this period. The ads had a clear rationale for semi-nudity: concentrated entirely in the summer months and aiming to show the positive effects of the product on the buyer’s entire body, depictions of bikini-clad bodies on beaches or by the pool made perfect sense. Often, they evoked a hazy ideal of Mediterranean heat and glamour—perhaps in line with the post-war rise in northern Europe of naturist tourism to France.^98^ However, it is notable that ads for tanning products did not usually appeal to (hetero)sexual attraction. With very few exceptions, these ads either showed a woman on her own, luxuriating in the sun, or a family group of two adults and two children.^99^ Ads did not usually feature couples,^100^ and the copy did not suggest that tanning made women more attractive *to others*. Rather, ads featuring solo women evoked self-contained sensuality. As one ad proclaimed, ‘It’s your summer and you’re here to enjoy it […] you’re free. But you’re beautiful!’^101^ Ads for tanning products claimed to enhance good looks, but implied that this was for women’s own enjoyment and pleasure.

Adverts for Nivea and Ambre Solaire sun tan oil exemplify this type of ad.^102^ Each shows a young white woman reclining and relaxed, eyes closed and face turned upwards, as she basks in the sunlight, baring an expanse of glowing golden skin. This is in contrast to the bikini-clad cover girls of *She* and women’s weeklies, who smiled brightly and met the camera’s gaze. In the 1969 Nivea advert, the model wears a bikini and turns her body towards the camera, carefully posing to show her throat and chest.^103^ She holds her knee, suggesting sensuous touch. At her feet are superimposed images of the Nivea product range, with bottles featuring reclining women and tanned young families playing on the beach. The text encourages consumers unused to foreign travel to buy the products at home as ‘these sorts of things can cost a lot more overseas’. Although on display, the skin shown here works to advertise the purpose of the product as a tanning agent for a ‘carefree continental tan’ (one Nivea product was called ‘Continental Sun Oil’) and emphasises that ‘Nivea has the power to make your skin more beautiful’. The product’s desirability is reinforced by the image of blissful and glamorous relaxation abroad.

In the 1970 Ambre Solaire ad (reproduced below), the model is naked.^104^ Shot from the side and carefully positioned so the chair mostly hides her bare flesh, the viewer actually sees less skin than on the Nivea model. Yet this sense of strategic concealment, and especially the strapline ‘When you take almost everything off, make sure you’re wearing Ambre Solaire’, draws attention to the model’s nudity. Again, sensuality and sexuality blur into each other. The ad emphasised time alone, abroad, sunbathing as ‘continentals have been … for many years’; presumably topless, sexually uninhibited and enhancing their tans with oil.^105^ Earlier and later Ambre Solaire ads echoed this language and imagery. A 1969 ad shouted, ‘Mediterraneans wear AMBRE SOLAIRE’, and riffed on taking ‘almost everything off’ over an image of a relaxed and dreamy model lying down, head resting on one arm while she caressed the other, her pulled-down bikini straps emphasising her naked back.^106^ A 1974 version, meanwhile, contained only the text ‘Just add the sun. When you take almost everything off, make sure you’re wearing Ambre Solaire’, next to an image of a woman’s naked and deeply tanned back, glistening in the sun, her small bikini briefs showing the cleft of her buttocks.^107^

**Figure:**
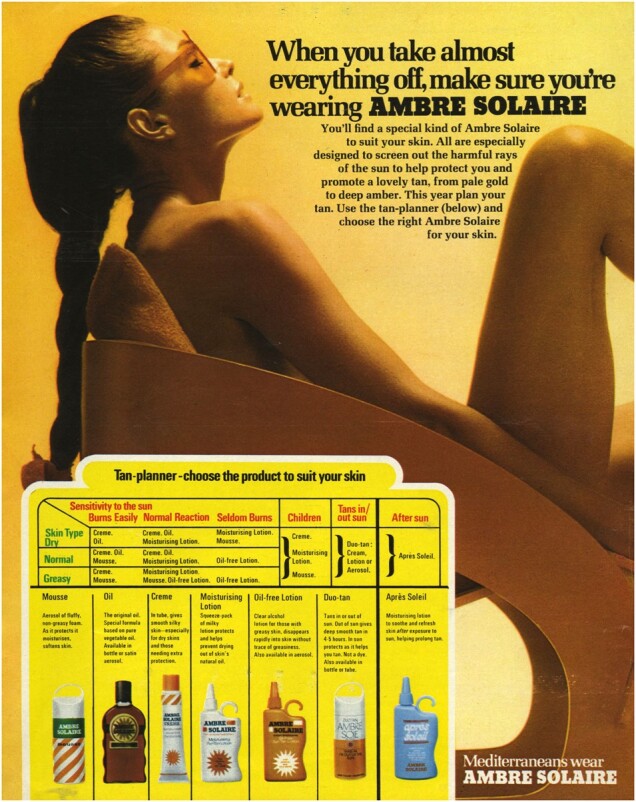
Image courtesy of The Advertising Archives

Most ads for tanning products evoked sensuality rather than explicit sexualisation. The exception is a 1971 series of Cooltan ads. One variant (reproduced below) showed a model’s back in close-up, her narrow waist contrasting with the curves of her bottom, and her low-slung bikini pants showing her buttock cleft.^108^ Her skin, darker than in most other tanning ads, glistened with sweat and/or oil. Somewhat queasily, the strapline read as a recipe: ‘Take a sensitive English skin, cover it with Cooltan, lay it under the sun, and turn slowly under golden brown’. Another ad in the series showed an aerial view of a white bikini-clad model lying on the sand, her tanned and oiled skin glistening with droplets from the sea.^109^ This image was cropped at clavicle and mid-thigh. These ads echoed Ambre Solaire’s innuendo, telling readers ‘when you take everything off—put Cooltan on’.

The Cooltan ads’ focus on ‘English skin’ highlights the construction of whiteness that is latent in other ads. Ads for many tanning products emphasised their suitability for use ‘under every sun, for every skin’, despite the uniform use of white models and text that assumed tanning patterns typical to Caucasian skin tones.^110^ More directly than other brands, the Cooltan ads explained that ‘English skin is fair and sensitive’, and that the brand ‘protect[ed] the English skin on holiday’ so users would ‘turn brown, not red’.^111^ Cooltan underscored the assumption of all ads for tanning products that the readers of these (British) magazines were English, and English consumers were white. Ads might portray the darker skin tones—the ‘golden glow’, ‘deep amber’ and ‘gentle golden brown’—that white people achieved through tanning as highly desirable, but naturally dark-skinned women were not welcome on magazine pages.^112^

The overt racialisation of the Cooltan ads existed alongside their overtly sexualised imagery. Unlike the ads discussed earlier, which sold the relaxation of a beach holiday, the Cooltan ads were singly focused on bared skin. The recipe-style strapline, combined with the dismembering of their bodies by the crop of the shots, reinforced the objectification of the models. The fact that these ads stand out, however, demonstrates the comparative rarity of highly sexualised and objectified nude images in women’s magazines. Nudity could be deployed in many ways, with the intention of provoking diverse responses among female viewers. If the very fact of the proliferation of nude images testifies to patriarchal visual culture in overdrive, then the content and context of images tell other, more complicated stories about the diverse potentialities of women’s sensual desires within and beyond sexual acts and behaviours.

**Figure:**
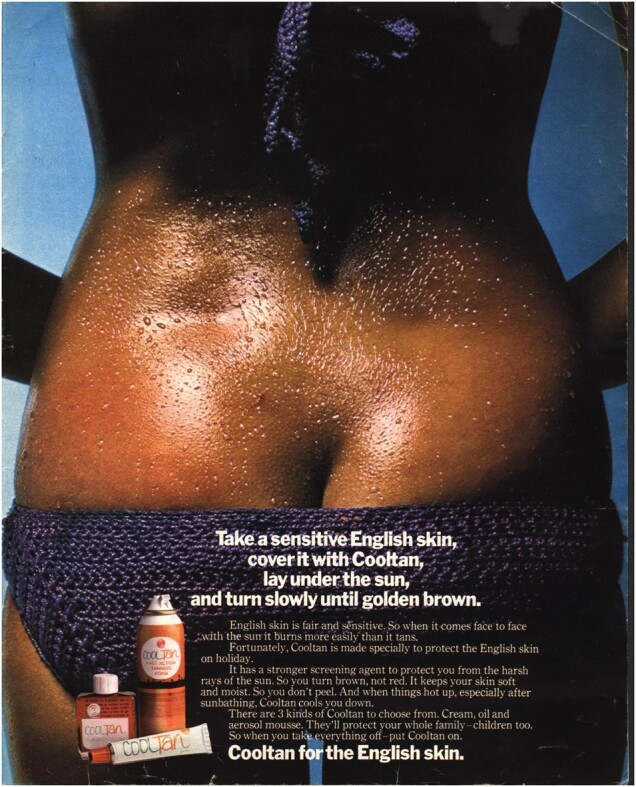
Image courtesy of The Advertising Archives

## Magazines, Reader Responses and Women’s Agency

So far, we have analysed these images more or less in isolation from their surrounding content within the magazine, focusing on the apparent intentions of advertisers and messages conveyed. Since the late 1980s, however, scholarship has emphasised that magazines are an ‘open-ended, heterogeneous, fragmented’ form.^113^ Magazines ‘harbour diversity, inconsistency, contradiction and tension’, partly because they mediate competing objectives, interests and viewpoints.^114^ Readers negotiate their encounters within these pages, employing different reading strategies, and actively make meaning out of what they seek and find—meanings that are shaped by the reader’s own experiences and intentions, as well as by the intentions of producers and the interaction of different types of content. The number of potential meanings is not endless, and nor are all potential meanings equally probable, not least because readers exist within the same cultural matrix, while editors seek to stimulate preferred readings for their ideal readership. But the fact remains: we cannot know exactly how each reader interpreted what she saw and read.^115^ The evidence does not exist. In this section, we place nude ads within the context of other relevant magazine content, including reader-generated content on related issues, to open out some of these possibilities.

Much scholarship has rightly underlined the power that advertisers held over magazine content. Mass-market magazines were never able to recoup costs from the cover price alone, and relied on advertising to generate revenue. Editors could not publish content that might alienate advertisers and put this income at risk, for example articles prominently featuring competitor products in issues that included costly advertising spreads from specific companies.^116^ By the early 1970s, advertisers also exerted positive influence over content. On 1 May 1971, *Woman* included a free sample of Femfresh vaginal deodorant, described as ‘from Helen Temple Beauty Editor’. The packaging included Temple’s signed endorsement in a form of wording jointly negotiated by the journalist, *Woman* publicity department, and Femfresh.^117^ The issue included a double-page colour advertisement for Femfresh, and an article by Helen Temple on deodorants. This recommended ‘vaginal deodorants made for a woman’s most sensitive area’ as ‘particularly useful during menstruation, hot days, and travelling’, and mentioned the free Femfresh sample. A line drawing of a nude woman with a rose motif covering her pubis and one breast illustrated the article. This floral imagery echoed advertisements for vaginal deodorants, which usually had floral rather than fruity scents as manufacturers wanted to avoid associations with taste and oral sex.^118^ The nefarious influence of advertisers on magazine content is evident here.

**Figure:**
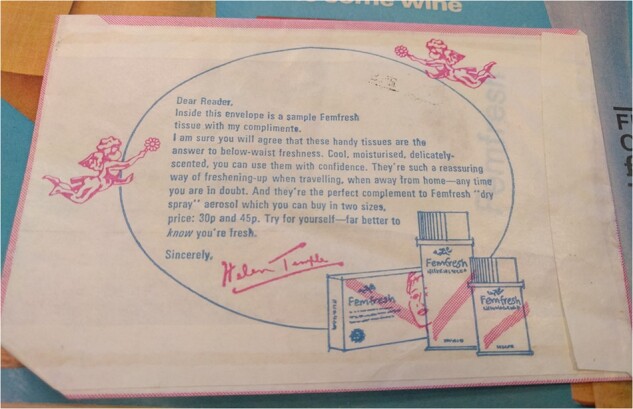
Image courtesy of The Walgreen Boots Alliance Archive

In general, however, the relationship between advertising and content across the magazine as a whole was more complex than in this example. All the magazines we consulted included a regular beauty column, organised around either topics like ‘winter beauty’ or ‘face exercises’, or products such as lipsticks or moisturisers. Articles on deodorants included discussion of vaginal deodorants, a product that readers encountered on the shelves of shops as well as in the pages of magazines. These discussions were broadly in step with the main messages of ads. An article from 1969 confirmed that vaginal deodorants only needed to be used during menstruation.^119^ In the early 1970s, when ads promoted everyday use, articles were more ambivalent. Columnists still described vaginal deodorants as most useful during menstruation, and diligently noted doctors’ views that they were unnecessary at any time, but added, ‘Our view is that if you don’t feel “clean” without one, then they’re as vital to you as teeth cleaning for all of us’.^120^ This stance might be read as either an abdication of responsibility or acknowledgment of women’s agency, but it certainly does not push manufacturers’ interests above conflicting perspectives. Moreover, even these ambivalent messages could be undercut elsewhere in the magazine. *Woman’s Own* health columnist Ruth Martin unequivocally stated that the ‘healthy vagina is self-cleansing’ and ‘women need do little more than follow a pattern of normal cleanliness’.^121^ Readers faced with different viewpoints might end up confused, but they could also assess what advice to follow based on the authority afforded to different magazine formats and columnists. The kaleidoscopic nature of magazines undercut the promotion of any single message.

We know little about how readers *did* respond to magazines. Reader-generated content is not a straightforward guide to readers’ opinions. Editors selected letters for publication to represent the magazine’s self-image as much as its readership, and edited published letters for length and coherence. Nevertheless, this material indicates how at least some readers responded to nude images, and how these responses were located within the nexus of gender and sexual relations. *She* and *Nova* actively courted readers’ views on nudity. Some letters were light-hearted and positive, as readers humorously reported on children calling *She* the ‘“Bum” book’, or the difficulty of finding a non-nude image when the boss unexpectedly turned up at her desk while reading the magazine.^122^ Others expressed embarrassment at feeling unable to read articles on the train without other passengers potentially passing judgment on the nudity in adverts.^123^*She* and *Nova* also printed forthright criticisms of sexualised covers that readers assumed were intended to attract men.^124^ These letters show that readers had noted ‘the current nude advertising craze’, and that some objected and related it to a wider proliferation of pornographic publications.^125^ Other readers, however, depicted those who complained about ‘bare breasts, bottoms and what-have-you’ as ‘small-minded, over-virtuous, super-hygienic inhuman women’.^126^ Less important, perhaps, than these differences of opinion is that magazines could keep such debates running over several issues as a way of pursuing their self-presentation as a ‘permissive’ forum for a younger, more educated audience.^127^

In contrast, the letters pages in women’s weeklies were deliberately light-hearted, sharing readers’ amusing stories, tips or wry reflections. Letters sometimes referred to trends associated with ‘permissiveness’, but only as off-the-cuff remarks—as from the ‘Pornogranny’ reporting her grandson’s comment that she had read him a ‘dirty book’ after she said his rag book needed washing.^128^ The tone and content of the page reflected deliberate editorial decisions not to engage with ‘serious’ issues in this forum. Editors of weeklies were cagey about responding to social transformations; they sought to strengthen reader loyalty through ostensibly non-hierarchical and community-building vehicles such as letters pages, but carefully policed these pages and avoided potentially controversial content. Readers’ letters pages in weeklies therefore do not shed much light on readers’ responses to ‘permissiveness’, sexual relationships or nudity. Rather, these issues were dealt with, but more obliquely because expressed via individual anxieties and fears, on the problem page. From these pages, we glean some sense of changes in readers’ lives, the negotiation of moral boundaries, and the gendered dynamics of sexual expectations and behaviour—on and off the page.

The problem pages in women’s weeklies reflect some of the social changes associated with ‘permissiveness’. More readers now wrote in admitting to premarital sex, not as an occasional slip, but as an established pattern within a relationship (albeit one that generated anxieties). Wives admitted sexual frustration, and in response agony aunts advised how to seduce their husbands.^129^ At the same time, agony aunts still saw marriage as the ultimate aim of relationships, portrayed sex as an unmitigated good only as ‘a symbol of love’ within matrimony and certainly did not encourage pre- or extramarital sex, even if they did not always condemn it.^130^ People might feel ‘tormented by sexual desire’ outside marriage, ‘just as a diabetic may long for sugar’, but those urges could be resisted.^131^ Moreover, assumptions about gendered roles and responsibilities reveal that power remained heavily skewed in favour of men. Letters from girls under pressure to have sex with boyfriends, or pregnant women abandoned by lovers, constructed an image of male sexuality as more urgent and less governable than female desire, of men as sexual aggressors, and of women as responsible for deflecting their advances.^132^ If husbands did *not* want sex, then women should take the initiative and not ‘carp’ as men found this off-putting.^133^ Similarly, women worried about their husbands’ wandering eyes were told how to artificially enhance their busts or lose weight.^134^ A woman who had burnt her husband’s stash of girlie mags was told that all men enjoyed this material, and that she needed to apologise and buy him some new ones.^135^ Moreover, so long as marriage was perceived as sacrosanct, women were expected to put up with disrespectful, misery-inducing or even dangerous behaviour, including violence.^136^ It was still perceived as women’s role to maintain emotional and sexual relationships—at all costs.

The ‘permissive’ monthlies also featured conservative attitudes to the body and sexual behaviour, in retrospect even more striking because articulated within a self-consciously ‘liberated’ outlook. *She* agony aunt Denise Robins told a woman who was ‘not mad about sex’ that her husband was ‘just young, and sex appeals to him’, and that she should ‘try and be more generous’ in her ‘love-making’.^137^ This response naturalised the husband’s demands, and instead called into question the writer’s lack of desire.^138^*She’*s articles recognised that women’s desire and pleasure were an important part of sex, but placed the onus on women to conjure heterosexual sex and romance, whether by keeping slim through sex or following steps to seduction.^139^ Similarly, under covers featuring women in provocative poses, topless with stockings on or corset-clad and brandishing a whip, *Nova* included plenty of articles about sex which sought to titillate.^140^ These included discussions of female pleasure, but journalists often concluded that society had ‘overemphasised the importance of sexual climax for women’.^141^ This might reassure women who felt inadequate because they did not identify with the lust displayed on ‘every ad in the underground, every magazine, every writhing book-cover’, but it also let men off the hook.^142^*Nova’*s approach to desire and responsibilities in heterosexual relationships is perhaps best illustrated by the feature on ‘How to undress in front of your husband’. This photo-guide to stripping instructed women how to please their ever-willing, sexually impatient husbands with their handicraft skills as well as their bodies. It could be cut out and turned into a handy flipbook ‘to amaze him while he waits’, the stripper propelled ‘into action’ with one flick of the woman’s wrist—even though context suggests that *Nova* believed this was one feat men were perfectly capable of achieving on their own.^143^

Looking at the world within the magazine reinforces the view that women did not share equally in the ‘sexual revolution’. But this does not mean they lacked agency. One potential response to objectifying images was activism. In 1970, the group Women in Media (WiM) formed to fight discrimination against women working the media, and effect change in media representations of women.^144^ Throughout the 1970s and beyond, WiM investigated sexist content across multiple media platforms, met with the Advertising Standards Authority (ASA) to discuss the stereotyping of women, and encouraged its wider membership to raise their concerns with the ASA.^145^ Their efforts coincided with and spurred on the efforts of WLM members to challenge the objectification of women. From March 1973, *Spare Rib* featured a column called ‘Sellout’, reprinting examples of sexist advertising. The same issue carried an ad for Epic Records showing a topless woman with the strapline ‘Play Me’; the editorial collective accepted the company’s money, but ran the ad under large text saying ‘This advertisement exploits women’.^146^ By 1980, the *Guardian* had introduced a similar column, ‘Naked Ape’, collecting examples of ‘male chauvinism’ from across the media.^147^ Sexism had not gone away, but women launched their own offensives against it, using the same media platforms that disseminated these images.

Quieter, less traceable and undoubtedly more common than activism, however, was the possibility of readers engaging with images against the grain of their producers’ apparent intentions. In July 1969, a reader argued that *She* was ‘obviously ruled by men (or lesbians)’ because it included so many images of naked women. In response, the editorial team affirmed its heterosexuality, stating that none of them ‘writes Greek poetry or shows any other sign of deviating from the norm’.^148^ This part-jokey, part-defensive response reaffirmed the magazine’s ‘permissiveness’, but also its heterosexual flavour. However, such assertions could be challenged. In 1972, the lesbian newsletter *Sappho* put forward its own queer reading of magazine images featuring two women as ‘pseudo-gay scene[s]’, showing that lesbian women could and did interpret such images as appealing to their own desires.^149^ At other times, the editors took issue with the idea that visual culture particularly stimulated lesbians, but also reacted against heterosexual women speaking for them. In 1975, they objected to the claim of Mary Stott, editor of the *Guardian* women’s page and member of WiM, that ‘the desirable girls featured on the covers of women’s magazines’ were definitely *not* ‘there to stimulate lesbian longings in readers’. *Sappho’*s editors resented lesbians becoming pawns in the critique of magazine nudity, and reminded Stott that ‘lesbians are not funny-shaped men but women’.^150^ This evidence confirms that within the kaleidoscopic form of the magazine, each image constituted only one fragment of a shimmering tessellation that each reader, rotating the cell as she chose, rearranged into new patterns. The same pieces of glass could be almost endlessly rearranged, recomposed, shaken up—queered, even.

## Conclusion

In the nude images that proliferated in women’s magazines at the turn of the 1970s, we can trace the intersection of dominant ideas about whiteness, heterosexuality and femininity. White heterosexual women were simultaneously power/less; socially dominant by virtue of ethnicity and sexual orientation, they were also always culturally other and subordinate to the male, even where class complicated social relations. Representations of naked and partially naked female bodies in mass culture highlight the ambivalence of this status. The explosion of nude images speaks to changing social mores: the representation of naked bodies in such outlets would not have been possible a decade previously. But it does not speak to changing power relations between women and men, or between white heterosexual women and their diverse ‘others’. The nude bodies of white women were depicted within the normative framework of heterosexuality. To this extent, and when set against the absence of non-white and overtly queer female bodies, the abundance of images of white heterosexual women is evidence of cultural power. At the same time, nakedness often connotes vulnerability. An excess of images of *naked* female bodies, in at least some contexts, demonstrates the objectification of women denied the full subjecthood/subjectivity of males in a mass culture which is not only heterosexual, but also patriarchal.

In these images, white heterosexuality remains the assumed norm, but women remain the assumed object. However, meaning is dependent on context, and there is always room for more than one meaning. Although women’s magazines undoubtedly replicated dominant structures of gendered power, they also engaged seriously with women’s multifaceted lives and emotions. Readers’ letters praised magazines for providing ‘very knowledgeable advice’, including some from older women who recalled times when ‘*no* weekly or monthly magazine printed anything about sex’ and felt they ‘could have been spared countless worries if the printed word had made sex and so forth accessible’.^151^ This echoed *She’*s editorial line: ‘lots of people need help in learning about sex, conquering difficulties and beating that downward plunge of bad-sex, unhappy life, worse sex, misery. That’s why we have one or two articles about sex in each issue.’^152^ Women’s magazines proffered knowledge and advice that reflected the power hierarchies of the surrounding world, but could also offer a valuable counterpoint to prevailing trends, through the simple act of acknowledging and representing women’s concerns.

Crucially, when women began to fight back against such images in the early 1970s, their activism came from within the world of magazines, and recognised the power of such magazines to influence their readers’ lives: the journalists who formed WiM and the WLM activists who founded *Spare Rib* had that much in common. However, activism was not the only possible way to challenge visual culture. Although it is only one fragment of evidence, *Sappho’*s queer reading of advertisements underlines that once an image is out in the world, many gazes might rest upon it. In attributing power to one particular gaze, we recognise power relations, but may also unwittingly reinforce them. The feminist scholars who asserted women’s powerlessness in negotiating patriarchal visual culture demonstrated their *own* ability to resist this culture by critiquing it from outside, while denying the same agentic capacities to the readers of women’s magazines. Here, we prefer to end by asserting the provisionality of any interpretation, including ours. In this way, we not only reinstate the dynamic relationship between images inert on the page before *us*, and how millions of nameless past readers responded in unknowable ways to what was in front of *them*, but also refuse to collude in denying the full selfhood of those readers.

## Footnotes

1 Marcus Collins, *Modern Love: An Intimate History of Men and Women in Twentieth-Century Britain* (London: Atlantic Books, 2003), 134–63.

2 Our sample includes ads for all vaginal deodorant, bathing and tanning products from all issues of the monthlies *Nova* and *She* published between 1968 and 1972, and January, April, July and October issues of the weeklies *Woman* and *Woman’s Own* published in the same period.

3 Kenneth Clark, *The Nude: A Study of Ideal Art* (London: John Murray, 1956), 1.

4 John Berger et al., *Ways of Seeing* (Harmondsworth: Penguin, 1972), 53–60.

5 Philippa Levine, ‘Naked Truths: Bodies, Knowledge, and the Erotics of Colonial Power’, *Journal of British Studies*, 2013, 52, 5–25, quotation 9.

6 Women of colour were rarely represented in mainstream mass-market magazines in the UK in this period. When pictured, they were usually included in showbiz and gossip pieces, centring either on US-based singers and groups like Diana Ross and the Supremes, or on girlfriends and wives of white male celebrities, in pairings such as Yoko Ono and John Lennon or Shakira Basch and Michael Caine; or in holiday and ‘white saviour’ narratives, where they existed in the background to demonstrate a generalised exoticism. There is much to be said about the construction of whiteness through absent non-white ‘others’ in mass-market women’s magazines in this period, but the topic is too complicated to tackle here. We hope to develop these ideas more fully in a subsequent publication.

7 Claire Langhamer, ‘“Who the Hell Are Ordinary People?” Ordinariness as a Category of Historical Analysis’, *Transactions of the Royal Historical Society*, 2018, 28, 175–95.

8 One reviewer implied an overtly sexualised interpretation of all ads in the tanning and bathing sections; another referred specifically to the Ambre Solaire ad first printed in 1970 and reproduced here.

9 Catherine McCormack, *Women in the Picture: Women, Art and the Power of Looking* (London: Icon Books, 2021), 105.

10 Sarah Browne, *The Women’s Liberation Movement in Scotland* (Manchester: MUP, 2014), 11–20; Jonathan Moss, *Women, Workplace Protest and Political Identity in England, 1968-85* (Manchester: MUP, 2019), 56–81.

11 Sam Brewitt-Taylor, ‘Christianity and the Invention of the Sexual Revolution in Britain, 1963-1967’, *Historical Journal*, 2017, 60, 519–46, 528.

12 See Ben Mechen, ‘“Instamatic Living Rooms of Sin”: Pornography, Participation and the Erotics of Ordinariness in the 1970s’, *Contemporary British History*, forthcoming: https://doi.org/10.1080/13619462.2022.2051486.

13 Hera Cook, *The Long Sexual Revolution: English Women, Sex, and Contraception 1800-1975* (Oxford: OUP, 2004), 293. H.G. Cocks, ‘Saucy Stories: Pornography, Sexology and the Marketing of Sexual Knowledge in Britain, c. 1918-70’, *Social History*, 2004, 29, 465–84, 465. Adrian Bingham, ‘Pin-Up Culture and Page 3 in the Popular Press’, in Maggie Andrews and Sallie McNamara, eds, *Women and the Media: Feminism and Femininity in Britain, 1900 to the Present* (New York, London: Routledge, 2014), 192.

14 Ben Mechen, ‘Everyday Sex in 1970s Britain’ (unpublished PhD thesis, University of London, 2016), 31.

15 Angela Carter, ‘Truly It Felt Like Year One’, in Sara Maitland, ed., *Very Heaven: Looking Back at the Sixties* (London: Virago, 1988), 209–16, 214; Anna Coote and Beatrix Campbell, *Sweet Freedom: The Struggle for Women’s Liberation* (London: Picador, 1982), 19–20.

16 See, for example, Joyce Brothers, ‘Should You Undress in Front of the Children’, *Woman’s Own* [hereafter *WO*], 12 July 1969.

17 Simon Szreter and Kate Fisher, *Sex Before the Sexual Revolution: Intimate Life in England, 1918-1963* (Cambridge: CUP, 2010), 64. Kate Fisher, ‘'She Was Quite Satisfied with the Arrangements I Made': Gender and Birth Control in Britain 1920-1950’, *Past and Present*, 2000, 169, 161–93,165.

18 Szreter and Fisher, *Sex Before the Sexual Revolution*, 111.

19
*Ibid*., 90–1.

20 Angela Davis, ‘Oh No, Nothing, We Didn’t Learn Anything’: Sex Education and the Preparation of Girls for Motherhood, c.1930–1970’, *History of Education*, 2008, 37, 661–77, 661, 667, 670; Tanya Evans, ‘Knowledge and Experience: From 1750 to the 1960s’, in Sarah Toulalan and Kate Fisher, eds, *The Routledge History of Sex and the Body: 1500 to the Present* (London, New York: Routledge, 2013), 263.

21 Joan Malleson, *Any Wife or Any Husband: A Penguin Handbook* (Harmondsworth: Penguin, 1962), 110.

22 Cartwright, cited in Elizabeth Wilson, *Only Halfway to Paradise: Women in Postwar Britain, 1945-1968* (London: Tavistock Publications, 1980), 97.

23 Chelsea Saxby, ‘Towards a History of the Urinary Infection Club: Cystitis, Self-Help, and Experiential Expertise in 1970s Britain’ (unpublished paper, 3^rd^ Annual Congress of Northern Network for Medical Humanities Research, 23–24 January 2020).

24 Jenny Diski, *The Sixties* (London: Profile Books, 2010), 60–1.

25 Cook, *The Long Sexual Revolution*, 3. Only in 1991 did a landmark judgement assert that marriage vows did not denote automatic consent to sex, while marital rape was not explicitly designated illegal until 2003.

26 See, especially, Claire Langhamer, *The English in Love: The Intimate Story of an Emotional Revolution* (Oxford: OUP, 2013).

27 Caroline Rusterholz, ‘“You Can’t Dismiss that as Being Less Happy, You See It Is Different”: Sexual Counselling in 1950s England’, *Twentieth Century British History*, 2019, 30, 375–98, 391–5.

28 Hannah Charnock, ‘Teenage Girls, Female Friendship and the Making of the Sexual Revolution in England, 1950-1980’, *Historical Journal*, 63, 2020, 1032–53.

29 Bingham, ‘Pin-Up Culture’, 185–86, 188.

30 Frank Mort, *Capital Affairs: London and the Making of the Permissive Society* (New Haven and London: Yale University Press, 2010), 266–67.

31 Annebella Pollen, *Nudism in a Cold Climate: The Visual Culture of Naturists in Mid-20^th^-Century Britain* (Los Angeles: Atelier Éditions, 2021), 143.

32 Bingham, ‘Pin-Up Culture’, 190; Cook, *The Long Sexual Revolution*, 293.

33 Collins, *Modern Love*, 140–41, 150–52, 156–59. On the ‘new pornography’, see also Mort, *Capital Affairs*, 277–78.

34
*Ibid*.

35 Bingham, ‘Pin-Up Culture’, 195–6.

36 John Berger et al., *Ways of Seeing*, 46–7.

37 Laura Mulvey, ‘Visual Pleasure and Narrative Cinema’, *Screen*, 175, *16*, 6–18, 11, 13.

38 Coote and Campbell, *Sweet Freedom*, 207; Marsha Rowe, ed., *Spare Rib Reader* (Harmondsworth: Penguin, 1982), 25.

39 Ros Ballaster *et al*., *Women’s Worlds: Ideology, Femininity and the Woman’s Magazine* (Basingstoke: Macmillan, 1991), 37–8.

40 Edward Snow, ‘Theorizing the Male Gaze: Some Problems’, *Representations*, 1989, 25, 30–41, 31.

41 Caroline Evans and Lorraine Gamman, ‘The Gaze Revisited, or Reviewing Queer Viewing’, in Paul Burston and Colin Richardson, eds, *A Queer Romance: Lesbians, Gay Men and Popular Culture* (London: New York and Routledge, 1995), 13–56, 16.

42 Jennifer Evans, ‘Seeing Subjectivity: Erotic Photography and the Optics of Desire’, in Jennifer Evans, Paul Betts and Stefan-Ludwig Hoffman, eds, *The Ethics of Seeing: Photography and Twentieth Century German History* (Berghan: New York, Oxford, 2018), 182–204, 183.

43 Ballaster, *Women’s Worlds*, 111.

44 Janice Winship, *Inside Women’s Magazines* (London and New York: Pandora Press, 1987), 39–41.

45 Tracey Loughran ‘“The Most Helpful Friends in the World”: Letters Pages, Expertise and Emotion in British Women’s Magazines, c. 1960-1990’, in Laurel Forster and Joanne Hollows, eds, *Women’s Periodicals and Print Culture in Britain, 1940s-2000s: The Postwar and Contemporary Period* (Edinburgh: Edinburgh University Press, 2020), 133–49.

46 Ballaster, *Women’s Worlds*, 111.

47 Peggy Makins, *The Evelyn Home Story* (Glasgow: Fontana/Collins, 1975), 170.

48 Cynthia White, *Women’s Magazines 1693-1968* (London: Michael Joseph, 1970), facing 324.

49
*Ibid*., 217.

50 ‘Letter from the Editor’, *Woman*, 5 July, 69; White, *Women’s Magazines*, 232.

51 White, *Women’s Magazines*, 166.

52 Winship, *Inside Women’s Magazines*, 46–7.

53 White, *Women’s Magazines*, 166.

54 Winship, *Inside Women’s Magazines*, 45.

55
*Nova*, March 1965.

56 Hera Cook, ‘Nova 1965-1970: Love, Masculinity and Feminism but Not as We Know It’, in Alana Harris and Timothy Willem Jones, eds, *Love and Romance in Britain, 1918-1970* (Basingstoke: Palgrave Macmillan, 2015), 226.

57 White, *Women’s Magazines*, 167, 224.

58 Excluding advertising, such images were most likely to be included in features on beauty, fashion, health and holidays, or on the cover.

59 For further discussion of vaginal deodorant advertising, see Daisy Payling, ‘Selling Shame: Feminine Hygiene Advertising and the Boundaries of Permissiveness in 1970s Britain’, *Gender & History*, first published online June 2022: https://doi.org/10.1111/1468-0424.12617.

60 Sara Ahmed*, The Cultural Politics of Emotion* (Edinburgh: EUP, 2004), 104.

61 Femfresh, *Woman*, 3 July 1971;*WO*, 17 July 1971.

62
*Nova*, July 1968; *WO*, 13 July 1968.

63
*WO*, 11 July 1970.

64
*WO*, 6 July 1978.

65
*She*, July 1970; *WO*, 11 April 1970; *Woman*, 25 April 1970.

66 Elspeth Probyn, *Blush: Faces of Shame* (Minneapolis, London: University Of Minnesota Press, 2005), xii.

67
*She*, April 1970; alternate version, *WO*, 9 October 1971. This strapline echoed a popular earlier ad for Lifebuoy soap that warned ‘What even your best friend won’t tell you’.

68 Constance Classen et al., *Aroma: The Cultural History of Smell* (London, New York: Routledge, 2002), 183.

69
*She*, April 1970; *Woman*, 17 April 1971.

70 Elle, *She*, July 1970; Femfresh, *WO*, 31 October 1970.

71
*WO*, 31 October 1970.

72
*She*, July 1971; *Woman*, 3 July 1971; *WO*, 17 July 1971.

73 A 1973 ad promised that Femfresh would ‘go on working as long as you do’, while one from 1974 argued that apart from a shower, Femfresh was ‘the most important thing in a working girl’s life’. *Woman*, 28 April 1973; *WO*, 14 April 1973.

74
*She*, July 1971.

75
*ibid*., July 1972.

76 For a similar theme, see Elle, *WO*, 3 July 1971.

77 Elle, *WO*, 11 July 1970.

78
*WO*, 27 January 1968; see also Bidex ads, *WO*, 6 July 1968, 27 July 1968, 5 July 1969, 12 July 1969, 19 July 1969, 26 July 1969.

79 See, for examples, FDS, *WO*, 27 July 1968, and ads for Fresh ‘n’ Dainty: *Woman*, 19 April 1969; *WO*, 6 April 1968, 5 July 1969, 17 January 1970.

80 Mimosept, *WO*, 25 July 1970; Mimospray, *WO*, 22 July 1972; FDS, *Woman*, 8 July 1972.

81
*Woman*, 3 April 1971, 3 July 1971; *WO*, 3 October 1970, 10 April 1971.

82 Concern, *WO*, 4 July 1970.

83 Brian Harrison, *Finding a Role? The United Kingdom, 1970-1990*, (Oxford: OUP, 2010), 18.

84 Sina Fabian, ‘Flight to the Sun: Package Tours and the Europeanisation of British Holiday Culture in the 1970s and 1980s’, *Contemporary British History*, 2021, 35, 417–38, 423–24.

85 Sue Wright, ‘Sun, Sea, Sand and Self-Expression: Mass Tourism as an Individual Experience’, in Hartmut Berghoff et al., eds, *The Making of Modern Tourism: The Cultural History of the British Experience, 1600-2000* (Palgrave Macmillan: Basingstoke, 2002), 181–202, 181.

86 Glen O’Hara, *The Politics of Water in Post-War Britain* (Basingstoke: Palgrave Macmillan, 2017), 183–214.

87
*Nova*, July 1970; *She*, July 1970; *Woman*, 4 July 1970.

88 Claire Hickman, ‘Pine Fresh: The Cultural and Medical Context of Pine Scent in Relation to Health—From the Forest to the Home’, *Medical Humanities*, 2021, 0, 1–10, 4.

89
*Nova*, January and April 1969.

90
*WO*, 20.4.68. *Venus pudica* describes a pose in which a nude female self-consciously covers her groin from sight. McCormack, *Women in the Picture*, 44,

91
*Woman*, 11 October 1969.

92
*WO*, 19 October 1968.

93
*She*, July 1970.

94
*WO*, 5 July 1969.

95 Jacqueline Sarsby, *Romantic Love and Society: Its Place in the Modern World* (Harmondsworth: Penguin, 1985), 70–98; Helen Taylor, ‘Romantic Readers’, in Helen Carr, ed., *From My Guy to Sci-Fi: Genre and Women’s Writing in the Postmodern World* (London: Pandora Press, 1989), 58–77.

96
*Nova*, April 1970, July 1970, April 1971, October 1971, April 1973; *Woman*, 29 April 1972, 28 October 1972; *WO*, 28 April 1973.

97
*Woman*, 29 April 1972.

98 Stephen L. Harp, *Au Naturel: Naturism, Nudism, and Tourism in Twentieth-Century France* (Louisiana State University Press: Baton Rouge, 2014), 2.

99 For an example of a ‘family group’ ad, see Uvistat, *WO*, 13 July 1974.

100 The one identified exception to this rule is an ad for Nivea, *WO*, 11 July 1970.

101 Cooltan, 15 July 1972.

102
*She*, July 1969; *She*, July 1970; *Woman*, 25 July 1970.

103
*She*, July 1969; *WO*, 26 July 1969.

104
*She*, July 1970; *Woman*, 25 July 1970.

105
*She*, July 1970, July 1971.

106
*WO*, 17 July 1969.

107
*WO*, 13 July 1974.

108
*WO*, 3 July 1971.

109
*She*, July 1971.

110 Ambre Solaire, *Woman*, 6 July 1968; see also Skol, *Woman*, 11 July 1970, and Ambre Solaire, *Woman*, 25 July 1970.

111
*She*, July 1971.

112 Nivea, *WO*, 26.7.69; Ambre Solaire, *Woman*, 25 July 1970; Nivea, *WO*, 11 July 1970.

113 Ballaster, *Women’s Worlds*, 7.

114 Penny Tinkler, ‘Fragmentation and Inclusivity: Methods for Working with Girls’ and Women’s Magazines’, in Rachel Ritchie et al., eds, *Women in Magazines: Research, Representation, Production and Consumption* (New York and London: Routledge, 2016), 25–39, 31.

115 Joke Hermes, *Reading Women’s Magazines: An analysis of Everyday Media Use* (Cambridge: Polity Press, 1995), 31–65.

116 Winship, *Inside Women’s Magazines*, 40.

117 The Walgreens Boots Alliance Archive, WBA/BT/BH/CPD/1/22/19, Woman Magazine Femfresh Samples, 1971.

118 Elana Levine, ‘“Having a female body doesn’t make you feminine”: Feminine Hygiene Advertising and 1970s Television’, *The Velvet Light Trap: A Critical Journal of Film and Television*, 2002, 50, 39–58, 41.

119 Phyllis Digby Morton, ‘Nice to be Near’, *WO*, 12 July 1969.

120 Helen Temple, ‘Stay as Sweet as You Are’, *Woman*, 28 July 1973.

121 Q & A, *WO*, 5 October 1968.

122
*She*, July 1969, 43–5; *She*, October 1970, 54.

123
*She*, January 1976, 9.

124
*Nova*, January 1975.

125
*She*, July 1969, 43–5; *Nova*, April 1972,16.

126
*She*, January 1970.

127
*She*, January–April 1970.

128
*Woman*, 4 October 1969.

129
*Woman*, 27 April 1968; *Woman*, 9 January 1971.

130
*Woman*, 18 July 1970; see also 11 July 1970.

131
*Woman*, 10 October 1970.

132
*Woman*, 10 January 1970; *Woman*, 9 January 1971; *Woman*, 10 October 1970; *Woman*, 24 October 1970.

133
*Woman*, 6 July 1968; see also *Woman*, 4 October 1969.

134
*Woman*, 10 October 1970; see also *Woman*, 31 October 1970.

135
*Woman*, 12 July 1969.

136
*Woman*, 31 October 1970.

137
*She*, July 1969

138 The perspective shifted somewhat when Dr David Delvin’s sex column, which encouraged both partners to take responsibility for their sexual satisfaction and safety, was introduced in 1973. See, for example, *She*, April 1973.

139
*She*, January 1971; *She*, January 1972, 22–3.

140
*Nova*, January 1970, May 1971, January 1972.

141
*Nova*, January 1969.

142
*Nova*, October 1974.

143
*Nova*, January 1971. *Nova*, May 1971.

144 Josephine King and Mary Stott, eds, *Is This Your Life? Images of Women in the Media* (London: Virago, 1977), 1–3.

145 LSE, 6WIM/A/01/01, WiM Minutes, WiM Minutes, 31 July 1972; LSE, 6WIM/A/01/02, WiM Minutes, 26 February 1973; LSE, 6WIM/A/01/03, WiM Minutes, 25 November 1974; LSE, 6WIM/A/01/04, WiM Minutes, 27 October 1975.

146
*Spare Rib* 9 (March 1973), 9.

147 Andrew Veitch, ed., *Naked Ape: An Anthology of Male Chauvinism from the Guardian* (London: Gerald Duckworth & Co., 1981).

148
*She*, July 1969, 43–5.

149
*Sappho*, 1:2, 1972, 15.

150
*Sappho*, 4:2, 1975, 3.

151
*She*, April 1972.

152
*She*, January 1972,16–17.

## Notes

Daisy Payling is a Public Engagement Officer at Queen Mary University of London and Community Fellow at the University of Essex. Before this, Daisy was a research fellow at the University of Essex working on the ‘*Body, Self and Family: Women’s Psychological, Emotional and Bodily Health in Britain, c. 1960-1990*’ project. Her work explores gender, health and women’s magazines.

Tracey Loughran is author of *Shell-Shock and Medical Culture in First World War Britain* (CUP, 2017), co-editor (with Gayle Davis) of *The Palgrave Handbook of Infertility in History: Approaches, Contexts and Perspectives* (Palgrave, 2017) and editor of *A Practical Guide to Studying History: Skills and Approaches* (Bloomsbury, 2017). Her current research explores women’s psychological, emotional and physical health in Britain between the 1960s and the 1990s.

