# Correction

**DOI:** 10.1093/shm/hkae017

**Published:** 2024-03-27

Due to an editorial oversight, three articles in the current Special Issue, Sexpertise: Sexual Knowledge and the Public in the Nineteenth and Twentieth Centuries, were published in two previous issues, Vol. 36 Issue 3, and Vol. 35 Issue 4. They are:

Elodie Serna, ‘Vasectomy in Interwar Europe: From Medical to Political Practices’, *Social History of Medicine*, Volume 36, Issue 3, August 2023, Pages 456–471, https://doi.org/10.1093/shm/hkac052

Mónica García-Fernández, ‘“A Healthy Sex Life”: Love, Marriage and Sexual Knowledge in Franco’s Spain (1960–1975)’, *Social History of Medicine*, Volume 35, Issue 4, November 2022, Pages 1334–1355, https://doi.org/10.1093/shm/hkab132

Daisy Payling and Tracey Loughran, ‘Nude Bodies in British Women’s Magazines at the Turn of the 1970s: Agency, Spectatorship, and the Sexual Revolution’, *Social History of Medicine*, Volume 35, Issue 4, November 2022, Pages 1356–1385, https://doi.org/10.1093/shm/hkac032

The papers are linked here to help readers see all papers in this collection together. The publisher apologizes for the error.

